# Soft tissue hemangioma of the right upper extremity with intraosseous extension and secondary intravascular papillary endothelial hyperplasia

**DOI:** 10.1007/s00256-024-04727-y

**Published:** 2024-07-12

**Authors:** Rachel Bass, Gene Siegal, Apoorva Kotha, Yulia Melenevksy

**Affiliations:** 1 Department of Radiology, University of Alabama at Birmingham, 619 19th St S, JTN 304, Birmingham, AL 35249 USA; 2 Department of Pathology, University of Alabama at Birmingham, 619 19th St. South, HSB 149K, Birmingham, AL 35249 USA; 3 Department of Radiology, Children’s Hospital of Philadelphia, 3401 Civic Center Blvd, Philadelphia, PA 19104 USA; 4 Department of Radiology, University of Alabama at Birmingham, 619 19th St S, JTN 342, Birmingham, AL 35249 USA

**Keywords:** Intravascular papillary endothelial hyperplasia, Hemangioma, Multicompartmental

## Abstract

Intravascular papillary endothelial hyperplasia (IPEH), also known as Masson’s tumor, is an uncommon exuberant form of organizing thrombus that may occur within a vessel, vascular tumor, or hematoma and may change the imaging appearance to mimic an aggressive process. It must be distinguished pathologically from angiosarcoma. They have been most commonly reported within superficial soft tissue tumors, and rapid growth and effect on bone are rarely described. We present a case of a patient with a soft tissue hemangioma with IPEH with intraosseous extension that presented with a pathologic fracture of her right humerus with an aggressive appearing osseous lesion. CT and MRI demonstrated a multifocal ill-defined soft tissue mass throughout the right upper extremity with underlying cortical tunneling and scalloping of the proximal humerus. Similar imaging findings were also present in the distal humerus and ipsilateral scapula and evolved during her hospitalization. Following percutaneous biopsy revealing hemangioma with features of papillary endothelial hyperplasia with intraosseous extension, the patient died in the ICU secondary to unrelated septic shock. Diagnosis was confirmed at autopsy. Primary and secondary IPEH have been generally characterized as well-defined solitary masses, most often in the superficial soft tissues. This case of a deep soft tissue hemangioma with type II IPEH, intraosseous extension, and imaging findings of regional multicompartmental involvement is very unusual. Reporting of this case in the literature should be beneficial for pathologic correlation with similar confounding masses as well as propose a possible mechanism for intraosseous extension of soft tissue hemangiomas.

## Introduction

Soft tissue hemangiomas are a commonly encountered vascular soft tissue mass in clinical practice, accounting for 7% of all soft tissue tumors [1]. Although these lesions have characteristic imaging features, they often require biopsy to exclude other benign and malignant entities. There are well-described reactive changes to the underlying bone including thickening or tunneling of the cortex, typically nonaggressive periosteal reaction, and erosions. On MRI, larger lesions are associated with underlying marrow edema [2–4].

Intravascular papillary endothelial hyperplasia (IPEH), also known as Masson’s tumor, may occur secondarily within a vascular mass such as a hemangioma (type II). It is an idiopathic intravascular proliferation associated with a vessel wall with a distinct histopathologic appearance, and is now regarded as an exuberant form of intravascular organizing thrombus [5]. Most reported cases are in the soft tissues, and presence within bone is rare and mostly documented in skull-based locations [6–8]. Occurrence within the appendicular skeleton is limited to a few case reports [9, 10]. The awareness of the diagnosis is important, as it has unique imaging and pathologic characteristics that may be confused pathologically for angiosarcoma.

We present a case of a soft tissue hemangioma of the upper extremity with intraosseous extension with a type II IPEH and pathologic fracture of the proximal humerus. The patient developed rapid growth of this mass by imaging throughout the upper extremity and chest wall along with osseous invasion, which has not been previously described in the English language literature.

## Case report

### History

A 69-year-old woman presented to the emergency department for several weeks of right upper extremity pain, swelling, and functional decline. She had been diagnosed at an outside hospital with a pathologic proximal right humeral fracture several weeks prior and was awaiting outpatient workup. The patient also had a notable recent medical history of right plasma cell mastitis and recurrent right pleural effusion of uncertain etiology 2 months prior to presentation. Upon admission, the patient was noted to have multiple lab abnormalities including leukocytosis (WBC 15.53 10^3^/cm) and thrombocytosis (520.3 10^3^/cm). She also had a large right pleural effusion with a pre-existing right pleural drain and multiple left pulmonary emboli.

### Imaging

**Fig. 1:**
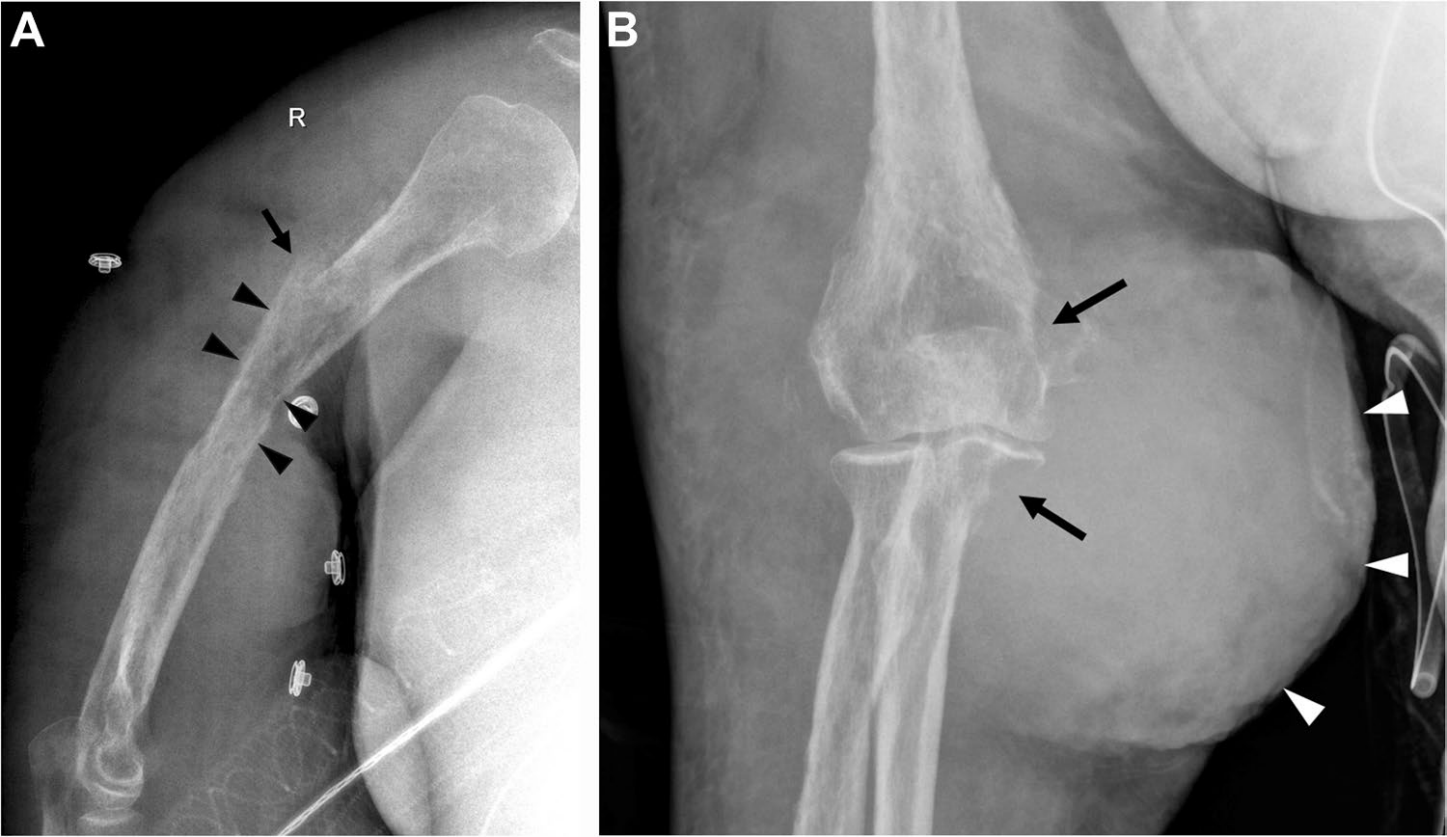
Lateral radiograph of the right humerus (**A**) demonstrates an aggressive permeative lesion of the right humerus (black arrowheads) with a pathological fracture (black arrow). AP radiograph of the elbow (**B**) shows aggressive lesions of the distal humerus and proximal ulna (black arrows) and large soft tissue mass medial to the elbow (white arrowheads)

Radiographs were obtained in the emergency department for upper extremity pain. There was a subacute pathologic fracture of the proximal and mid right humeral diaphysis with an underlying ill-defined permeative lesion with wide zone of transition and cortical thinning (Fig. 1). There were areas of cortical destruction at the elbow along the medial humeral epicondyle and proximal ulna with an associated overlying mass-like soft tissue swelling (Fig. 1).

**Fig. 2:**
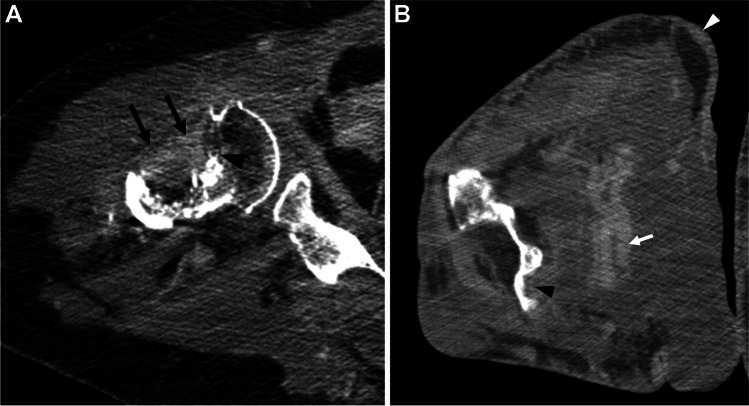
CT axial image at the level of the humeral head (**A**) shows area of cortical destruction (black arrowhead) with enhancing soft tissue (black arrows). At the level of the elbow (**B**), there are skin thickening (white arrowhead), tortuous blood vessels (white arrow), and erosion of the medial humeral condyle (black arrowhead)

CT with contrast of the right humerus was subsequently performed. An area of cortical destruction was present along the anterior inferior humeral head at the cranial margin of the pathologic fracture. There was an enhancing soft tissue mass that extended into the proximal humeral diaphyseal intramedullary space with associated endosteal scalloping and cortical thinning (Fig. 2). Below the level of fracture, there was circumferential cortical thinning and cortical tunneling with preserved marrow fat. Additional areas of cortical destruction with overlying soft tissue mass-like enhancement were present in the medial and lateral humeral epicondyle and proximal ulna. The musculature of the right upper arm was diffusely indistinct with ill-defined heterogeneous enhancement extending beyond fascial planes and approaching the skin surface, greatest at the level of the elbow. There was no surrounding edema or calcification.

**Fig. 3:**
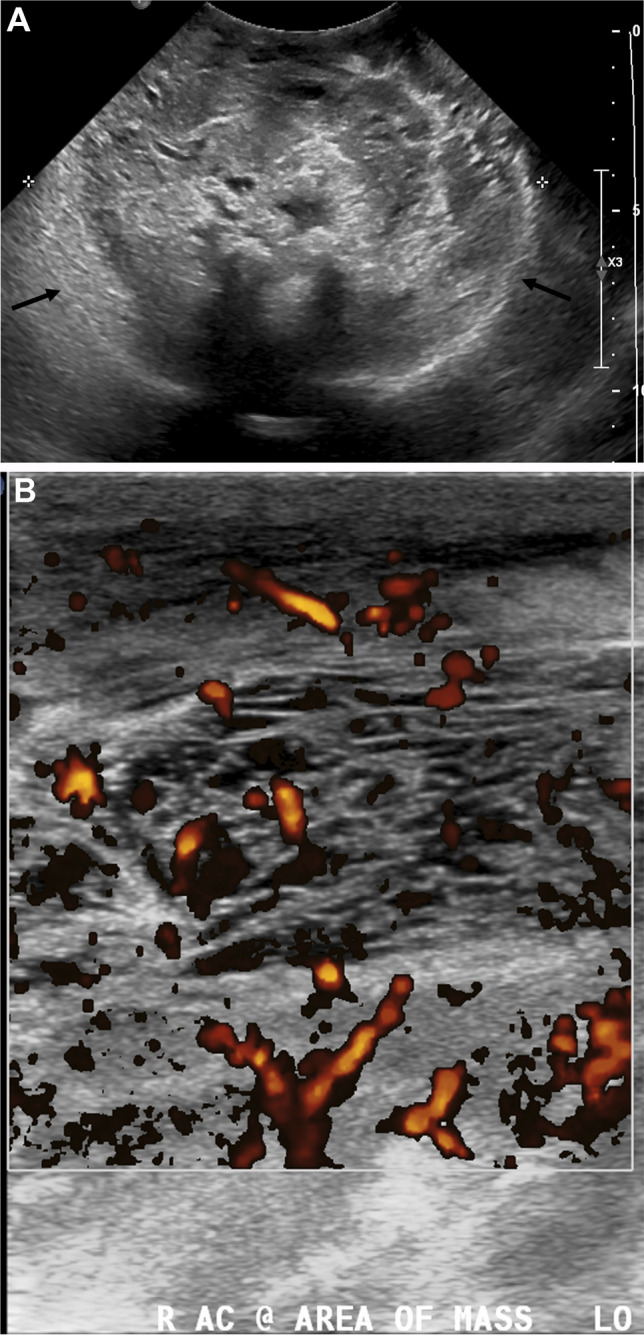
Gray scale (**A**) and Doppler (**B**) ultrasound images of the antecubital fossa demonstrate a large, heterogeneous, vascular mass (arrows in **A**)

A right upper extremity ultrasound examination was ordered for evaluation of deep vein thrombosis and demonstrated a heterogeneously hypoechoic, hypervascular mass replacing the muscle architecture with multiple anechoic, cystic areas with internal septations and dilated traversing vessels (Fig. 3). Intralesional lobular echogenic areas represented areas of thrombus and slow flow with vascular septations on color Doppler images.

**Fig. 4:**
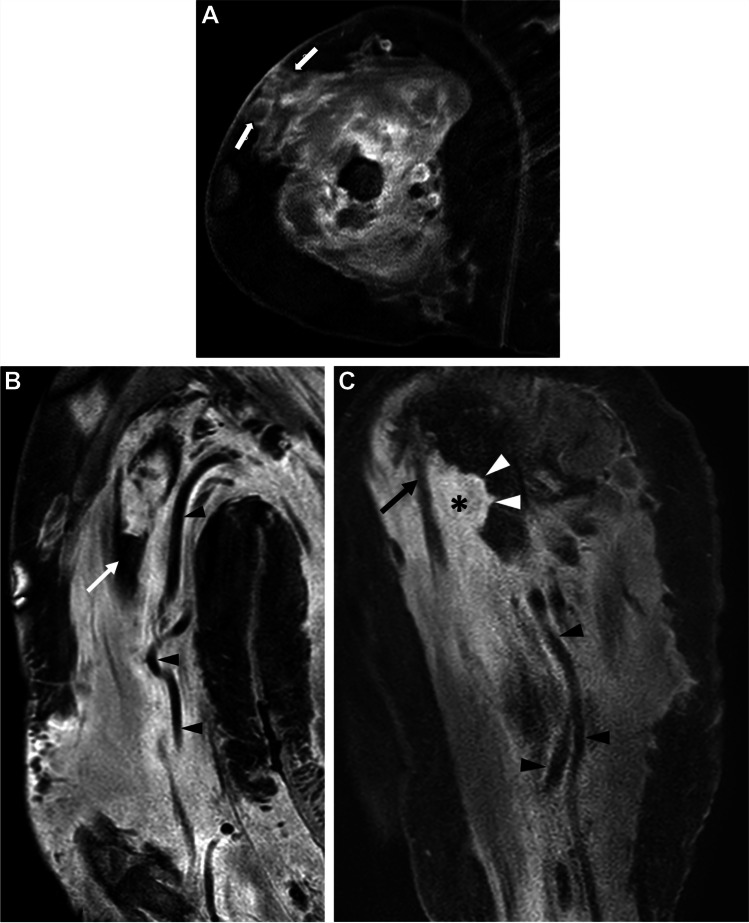
Axial post-contrast-enhanced T1W fat–saturated image of the upper arm (**A**) shows a heterogeneously enhancing mass extending to the skin (block arrows). Coronal contrast-enhanced T1W fat–saturated image of the upper arm (**B**) shows heterogeneous enhancement of the muscles with dilated, tortuous vessels coursing through the mass without compression (white arrowheads in **B** and **C**). Note preserved normal marrow signal distal to the lesion (white arrow). Sagittal post-contrast-enhanced T1W fat–saturated image of the upper arm (**C**) demonstrates an enhancing soft tissue (asterisk) with underlying cortical destruction (white arrowheads) and surrounding long head of the biceps tendon (black arrow)

MRI with contrast was performed for biopsy planning and was technically limited by extensive artifact on multiple sequences. Throughout the upper arm, there was extensive heterogeneous soft tissue enhancement with STIR hyperintensity with loss of the normal muscular architecture, extending to the skin surface and containing multiple dilated traversing vessels without stenosis (Fig. 4). There was increased enhancement within the area of proximal humeral destruction with intense intraosseous enhancement and STIR hyperintensity extending caudally to the fracture. The intramedullary marrow fat was preserved above and below the level of the fracture.

A CT-guided biopsy was performed of the enhancing soft tissue and bone at the level of proximal humeral cortical destruction proximal to the pathologic fracture. Notably, on the planning CT of the right shoulder, there was similar ill-defined musculature and mass-like area approaching the skin surface of the right shoulder girdle and chest wall which had markedly progressed in size since admission, as well as a new associated permeative appearance and pathologic fracture of the inferior right scapular body.

### Pathology

**Fig. 5:**
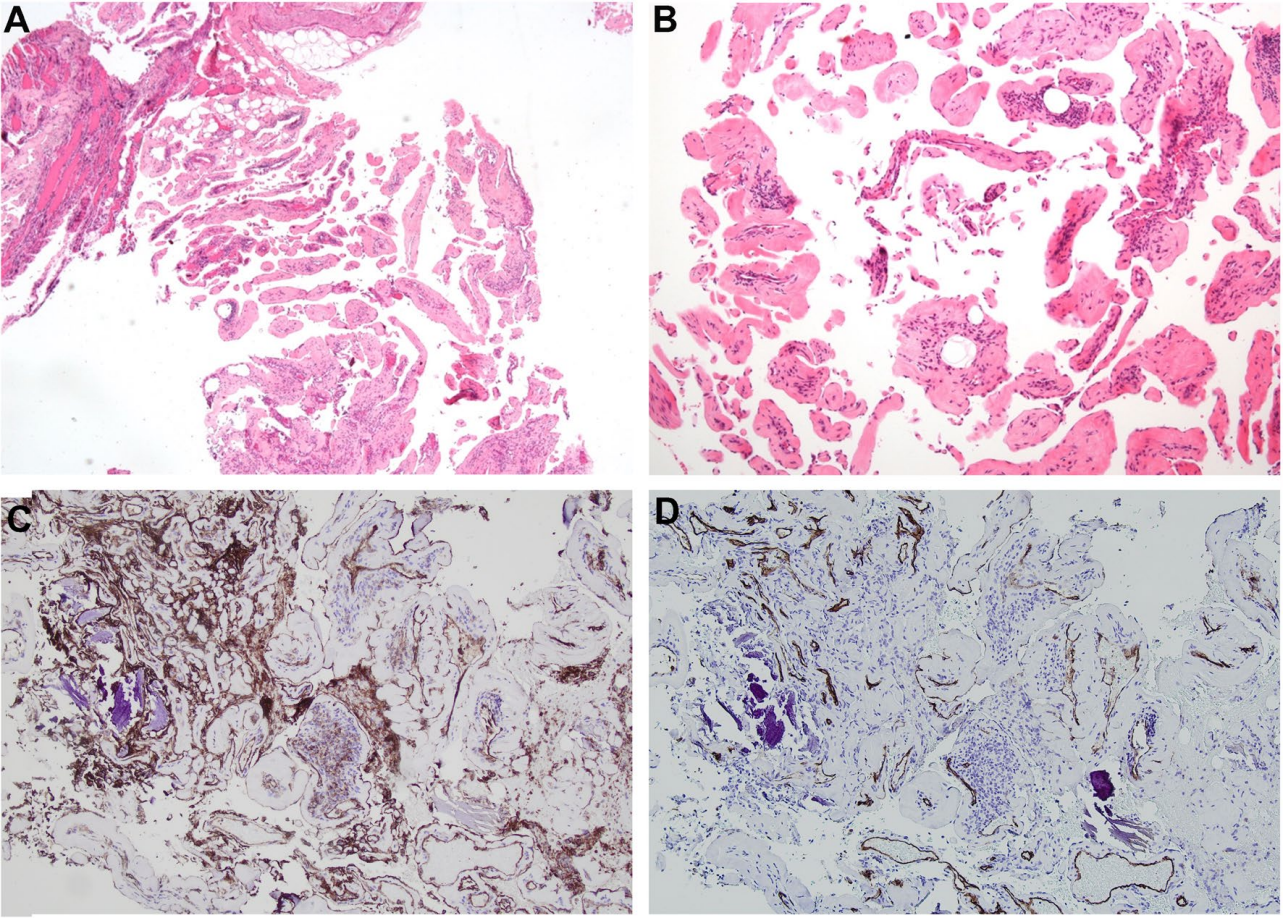
A Low power view reveals the lesion to be composed of innumerable “finger-like” projections composed of endothelial-lined vascular spaces enveloped by bland fibrous connective tissue and tiny fragments of striated muscle and adipose tissue at the edge [H & E, 40 ×]. **B** At higher power, tiny organized thrombi are appreciated in the center of the field. The papillary projections again demonstrate endothelial cells admixed with collagen cores and rare inflammatory cells filling the luminal space [H & E, 200 ×]. **C** Low power view with CD31 and **D** CD34 stains highlighting the endothelial cells in the mass [10 ×]

A core needle biopsy revealed the presence of a vascular mass with innumerable “finger-like” projections in the endothelial-lined vascular walls and a central organizing thrombus, which are features of intravascular papillary endothelial hyperplasia (Masson’s tumor). Endothelial cells were highlighted by CD31 and CD34 immunoreactivity. No cellular atypia was noted (Fig. 5). The final diagnosis on each separate biopsy of the humerus, the erosion, and the surrounding muscle was hemangioma with features of Masson’s tumor invading the surrounding bone.

The patient developed septic shock related to a pre-existing pulmonary infection and died in the ICU a week following her biopsy. Postmortem autopsy confirmed the presence of a 6.5 × 1.2 × 1.0 cm hemangioma within the humerus with thick-walled vessels and endothelial proliferation with hemorrhage in the growth pattern of an intravascular papillary endothelial hyperplasia. This was contiguous with a soft tissue hemangioma with same histopathologic features. The skin throughout the right chest wall and right upper arm was noted to be hyperpigmented, but not otherwise abnormal. Specific histologic examination of the soft tissues of the chest wall was not performed at the time of autopsy.

## Discussion

Hemangiomas are commonly diagnosed vascular tumors that describe a slow flow vascular malformation which occur primarily in the soft tissues or bone. Masses that are centered within the deep soft tissues and appendicular skeleton are more likely to be symptomatic and may become more ill-defined with increasing size. When superficial, they are often also accompanied by bluish discoloration of the skin.

The imaging features of soft tissue hemangiomas have been well-documented in the literature. Radiographic characteristics include internal calcifications and phleboliths with reactive changes to adjacent osseous structures including erosions, periostitis, and cortical tunneling [2–4, 11–15]. On ultrasound, they are heterogeneously hypoechoic with multiple cystic spaces and echogenic areas representing intralesional fat, phleboliths, or internal thrombi. While these masses are generally oval in shape, the margins may be irregular or ill-defined [16, 17]. On MRI, hemangiomas are classically hyperintense on T2-weighted images with intense enhancement and flow voids. T1 signal is variable due to interspersed fat and thrombus [1].

Intravascular papillary endothelial hyperplasia (IPEH) is a less common vascular soft tissue mass defined as a benign, exuberant proliferation of endothelial cells arising from a thrombus [5]. They may occur primarily within a vessel (type I) or secondarily within a vascular mass (type II) or extravascular within a hematoma (type III) [18]. These masses are most commonly identified in their primary form when they occur in superficial locations in the head, neck, and hands, and are generally well-circumscribed on imaging [19, 20]. In deeper locations, they often undergo biopsy to exclude more aggressive process. The proliferative appearance can be mistaken histologically for an angiosarcoma.

There is little in the radiology literature regarding the imaging features of type II IPEH. Lee et al. described two cases of type II IPEH in which the masses were well-defined and hypoechoic with internal vascularity, hyperechoic septa, and an echogenic central portion attributed to thrombus [18]. In the same study by Lee et al., the MRI characteristics of four cases of type II IPEH were described as isointense to hyperintense on T1 and T2 with foci of T1 hyperintensity and T2 hypointensity. In a single case, there was peripheral enhancement. Other sparse case reports of type II IPEH describe similar findings [18, 20, 21].

The presented case posed several imaging conundrums. To review, there was a pathologic proximal humeral fracture ill-defined deep and superficial soft tissue masses, cortical destruction, and cortical tunneling extending below the level of the humeral facture. On ultrasound, the soft tissue mass was hypoechoic and hypervascular with multiple cystic areas with enhancing septations. There were prominent traversing vessels within the soft tissue component on MRI, and the borders were ill-defined. The intraosseous component was hyperintense on T2 with enhancement. Below the fracture, there was preserved marrow fat with circumferential cortical thinning and tunneling.

The differential prior to biopsy primarily included malignant neoplastic and infectious processes. Angiosarcoma was considered given the intense enhancement, prominent vessels, and ill-defined borders. Lymphoma was also considered with its infiltrative borders, traversing vessels, and permeative appearance of the humeral cortex. However, the preserved marrow fat below the level of the pathologic fracture was confounding for this diagnosis. Osteomyelitis with infectious myositis was also considered, particularly in the setting of an elevated white cell count; however, the markedly abnormal imaging appearance of the musculature without evidence of an abscess or a periosteal reaction was felt to be unusual. The circumferential cortical thinning and periarticular scalloping and destruction also raised suspicion for an extensive, but low-grade neoplastic, fungal infection, or inflammatory process with pressure erosions.

Both the biopsy and autopsy verified that the proximal humeral osseous and soft tissue mass represented hemangioma. The pathologic fracture was at the level of the intraosseous extension of the hemangioma. Below the fracture, the circumferential cortical thinning and tunneling with preserved marrow fat corresponded to the reactive boney changes to the deep soft tissue hemangioma.

There are several features that make the final pathology of hemangioma with type II IPEH notable. First, the intraosseous involvement of this hemangioma is unique. Though we do not have prior imaging, the pathologic and radiologic consensus is that the mass originated in the soft tissues given the extensive soft tissue component and the relatively smaller osseous component. The literature describing the intraosseous extension of a soft tissue hemangioma is limited to a few case reports. One by Daoud et al. described a case of a soft tissue hemangioma with destruction and intraosseous invasion into the sacrum, with a proposed mechanism as possibly related to dysregulation of VEGF receptor expression [22]. This case proposes another possible mechanism for osseous destruction—superimposed IPEH, which may have inherently osteolytic properties. There are reported cases of IPEH occurring in the head neck which has resulted in underlying osseous destruction and erosion [6, 7, 9, 23]. Few case reports of primary intraosseous IPEH describe the lesions as osteolytic and expansile [10, 24–26]. It is proposed that in this case, the invasive nature of the soft tissue hemangioma into the proximal humerus and scapula could be attributed to the superimposed properties of the IPEH.

This case is also notable for the ill-defined to d borders and multicompartmental extension of this mass on imaging, which posed a diagnostic challenge. Interestingly, while IPEH is generally described as a well-defined mass, it can be ill-defined in the setting of a rupture vessel [5]. It is proposed that the pathologic humeral fracture caused the vascular disruption which may have led to more proliferative and ill-defined margins of the mass. This may also account for the rapid proliferation of the upper arm soft tissue findings to involve the shoulder girdle and ipsilateral chest wall was unusual. This unfortunately was not explicitly described at autopsy; however, given similar signal characteristics and accompanying skin discoloration, it is assumed to represent the same pathology as was found in the proximal humerus, namely hemangioma with IPEH. It is postulated that there would be a rapid increase in size of a secondary IPEH following trauma; however, antecedent trauma is only reported in 4% of cases of IPEH, so knowledge regarding its behavior in this setting may be lacking [27].

Secondary IPEH as a proposed mechanism of intraosseous extension of soft tissue hemangioma may contribute to the literature in the understanding of IPEH and consideration of this entity by musculoskeletal radiologists when there are mixed aggressive and nonaggressive features of an osseous lesion. The ill-defined margins and multicompartmental extension by imaging are highly unusual; soft tissue hemangioma is highly unusual and proposed to be related to the IPEH. It is the only case report in the English literature of its kind to the author’s knowledge.

## Data Availability

All authors declare that they had full access to all of the data in this study and the authors take complete responsibility for the integrity of the data and the accuracy of the data analysis.
